# Editorial: Bacterial Cell Wall Structure and Dynamics

**DOI:** 10.3389/fmicb.2019.02051

**Published:** 2019-09-04

**Authors:** Tobias Dörr, Patrick J. Moynihan, Christoph Mayer

**Affiliations:** ^1^Department of Microbiology, Weill Institute for Cell and Molecular Biology, Ithaca, NY, United States; ^2^Cornell Institute of Host-Microbe Interactions and Disease, Cornell University, Ithaca, NY, United States; ^3^School of Biosciences, Institute of Microbiology and Infection, University of Birmingham, Birmingham, United Kingdom; ^4^Department of Biology, Interfaculty Institute of Microbiology and Infection Medicine Tübingen, University of Tübingen, Tübingen, Germany

**Keywords:** peptidoglycan, cell wall, autolysin, PG recycling, turnover

The bacterial cell wall is a complex, mesh-like structure that in most bacteria is essential for maintenance of cell shape and structural integrity. Historically, the cell wall has been of intense research interest due to its necessity for most bacteria and absence from the eukaryotic realm, positioning it as an ideal target for some of our most powerful antibiotics (Schneider and Sahl, 2010). In addition, bacterial cell wall fragments can have immunostimulatory and cytotoxic properties and thus play important roles in pathogenesis and disease (Goldman et al., 1982; Fleming et al., 1986; Royet et al., 2011; Sorbara and Philpott, 2011; Jutras et al., 2019).

The cell wall consists mainly of peptidoglycan (PG), a mesh of polysaccharide strands (composed of a poly-[*N*-acetylglucosamine (GlcNAc)-*N*-acetylmuramic acid (MurNAc)] backbone) cross-linked via short peptide bridges attached to the MurNAc residues (Vollmer et al., 2008a). PG is synthesized on the external face of the cytoplasm. Synthesis steps include cytoplasmic generation of the lipid-linked disaccharide-pentapeptide precursor lipid II, translocation of lipid II to the outside of the cell by flippases (MurJ and/or Amj); and finally assembly of the cell wall by penicillin-binding proteins (PBPs) and Shape, Elongation, Division, and Sporulation (SEDS) proteins (Ruiz, 2008; Typas et al., 2011; Meeske et al., 2015, 2016; Cho et al., 2016; Taguchi et al., 2019). The assembly process can be further subdivided into polymerization of the GlcNAc-MurNAc-pentapeptide via glycosyltransferase reactions catalyzed by class A PBPs and SEDS proteins, and crosslinking of the peptide sidestems into a tight meshwork by class A and B PBPs and l,d-transpeptidases in a not (yet) fully-understood manner (Zhao et al., 2017). In addition, the PG mesh can be decorated with secondary cell wall polymers, such as wall teichoic acids (polyol-phosphate polymers) or capsule polysaccharides that are covalently attached to PG (Rajagopal and Walker, 2017). In the case of mycobacteria, layers of polysaccharides and long-chain lipids are added to the PG layer, making the cell wall structure even more complex (Jankute et al., 2015).

While the cell wall must be rigid enough to maintain high intracellular pressures and withstand environmental assaults, it also needs to be flexible enough to allow for cellular expansion. In addition to synthesis functions, the cell wall is thus also constantly broken down, turned over, and remodeled (Park and Uehara, 2008; Reith and Mayer, 2011; Mayer et al., 2019). This is accomplished by a poorly-understood, remarkable group of enzymes that collectively can cleave and/or modify a variety of PG structures. So-called “autolysins,” for example, are a functionally diverse group of enzymes that cut PG crosslinks (endopeptidases), peptide sidestems (amidases, carboxypeptidases), or the sugar backbone (muramidases, lytic transglycosylases) (Scheurwater et al., 2008; Vollmer et al., 2008b). PG-acetyltransferases “decorate” MurNAc backbone structures with acetyl residues, imparting increased lysozyme resistance (Moynihan and Clarke, 2011). l,d-transpeptidases (Mainardi et al., 2008) orchestrate d-amino acid (DAA) exchange reactions that can replace terminal d-Ala residues with a variety of alternative DAAs (Cava et al., 2011); this can be exploited to label PG with fluorescent compounds (Kuru et al., 2012). Many of these systems fulfill important functions such as daughter cell separation, sacculus expansion during growth, insertion of macromolecular trans-envelope protein complexes, and PG recycling (Scheurwater and Burrows, 2011; Vollmer, 2012; Johnson et al., 2013).

Due to its importance for bacterial survival and the many open questions concerning mechanistic details of synthesis and turnover, the cell wall remains at the center of a large number of active research programs. The last decade in particular has seen a resurgence in interest in the bacterial cell wall—a Pubmed search with the keywords “peptidoglycan synthesis bacteria” reveals a total of 7,762 publications, of which 3,532 (45%) were published in the last 10 years. This renewed interest has been fueled by novel imaging techniques (super-resolution imaging and the development of live cell wall stains) and by new revelations of processes that had been thought to be well-understood for decades. Some recent examples include the finding that the class A “penicillin-binding proteins” (aPBPs) require outer membrane cofactors for *in vivo* function (Paradis-Bleau et al., 2010; Typas et al., 2010), and that RodA/FtsW possess glycosyltransferase activity (Taguchi et al., 2019). At the same time, the cell wall remains a highly attractive target for antibiotic development, which has in the last decade become ever more important due to the rise in antibiotic resistance development.

In this special topic issue, we explore some new developments in the realm of bacterial cell wall biology. This collection of articles touches upon several cornerstones of PG research, with contributions focusing on the cell wall as a target for novel antibiotics, and aspects of its synthesis, turnover and modification.

## The Cell Wall as a Target for Antibiotic Discovery

In a bioinformatics tour-de-force, Jukič et al. describe novel inhibitors of UppS, an isoprenyl transferase enzyme that catalyzes a critical step in the biosynthesis of the lipid carrier molecule undecaprenol pyrophosphate (UPP). UPP is essential for the translocation of the PG precursor lipid II and other extracellular polysaccharides and thus constitutes a promising target for a novel class of cell envelope antibiotics. These inhibitors were identified by virtual docking models that predicted molecule binding based on UppS crystal structures and their interaction with a known inhibitor, bisphosphonate BPH-629. This clever approach resulted in the identification of several inhibitors, one of them with μM range inhibitory activity.

Drug-resistant *Mycobacterium tuberculosis* strains are a major global threat that is not being adequately met with current drug discovery efforts. In their review article, Catalão et al. describe the history of peptidoglycan-targeting drugs and their use in mycobacteria. The authors provide a potential path forward by discussing recent advances such as therapies using β-lactam/β-lactamase inhibitor combinations and the use of phage endolysins for the treatment of mycobacterial infections.

## Cell Wall Synthesis and Architecture

Using X-ray crystallography and liquid state NMR, Maya-Martinez et al. investigate the structure-function relationships of PBP4 of *Staphylococcus aureus*, a class C PBP that unexpectedly has no PG hydrolase (d,d-peptidase) activity, but only transpeptidase activity. *S. aureus* is characterized by a very high degree of PG cross-linking and PBP4 apparently plays a major role in this hyper-crosslinking. The authors show transpeptidase activity of PBP4 with disaccharide peptides *in vitro*, producing dimeric, multimeric, and cyclic products. Structural studies with an active site mutant (S75C) revealed potential binding sites for the donor and acceptor stem peptides involved in the transpeptidation reaction.

Hottmann et al. report on peptidoglycan metabolism in the oral Gram-negative pathogen *Tannerella forsythia* (Phylum *Bacteroidetes*). *T. forsythia* depends on an exogeneous supply of the cell wall sugar *N*-acetylmuramic acid (MurNAc), as it lacks genes generally essential for bacteria for *de novo* synthesis of the peptidoglycan precursor UDP-MurNAc. A pathway for the catabolism of MurNAc involving a MurNAc-6 kinase (MurK) and a MurNAc-6P hydrolase (MurQ etherase) was established in *T. forsythia*, which counteracts a proposed cell wall synthesis pathway that utilizes salvaged MurNAc from the medium. Accordingly, a mutant in *murK* exhibited increased tolerance to low external MurNAc concentrations, presumably since blocking MurNAc degradation enhances peptidoglycan precursor synthesis.

The exact *in vivo* architecture of PG is poorly understood. Li et al. used Atomic Force Microscopy (AFM) for a detailed study of PG architecture, particularly at the septum, in *B. subtilis*. Surprisingly, *B. subtilis* undergoes significant changes in thickness and overall cell wall architecture in different growth phases. Li et al. were also able to isolate and image septa at varying stages of completion, visualizing the PG dynamics of septal closure at high resolution.

In a thought-provoking perspective article, Vincent et al. present a hypothesis for the evolutionary origins of the unique mycobacterial cell wall through a series of horizontal gene transfers. They support their argument by observing the distribution of key cell-wall biosynthetic enzymes across the order, which suggests that the arabinogalactan components pre-date the outer membrane and virulence related lipids. In their article, the authors propose an experiment whereby the evolutionary origins of the leaflet could be tested by attempting to reconstruct the mycobacterial cell wall in an *Actinobacterium* that currently lacks this feature.

## Cell Wall Turnover and Modification

Duchêne et al. describe new phenotypes for endopeptidase mutants in *Lactobacillus plantarum*. The mechanisms of regulation and physiological functions of cell wall lytic enzymes are still poorly understood, particularly in non-model organisms. *L. plantarum* is an ideal system to study PG hydrolase phenotypes due to its relatively small number of PG lytic enzymes (a “mere” twelve!). The authors carefully dissect the cell biological consequences of the loss of *L. plantarum*'s endopeptidases and assign new putative functions to these enzymes. This study thus lifts the curtain on endopeptidase function in a Gram-positive non-model organism, which is of particular importance given the high level of redundancy of PG lytic enzymes in many model bacteria, which ordinarily makes gene-phenotype association difficult.

During cell wall turnover in the Gram-positive pathogen *S. aureus*, the MurNAc-GlcNAc disaccharide is released from PG by the major autolysin Atl and its components eventually reused for PG biogenesis. Kluj et al. report on the fate of this disaccharide, which is taken up and is concomitantly phosphorylated by a phosphotransferase system (PTS) transporter. In order to facilitate PG recycling, the product MurNAc-6P-GlcNAc is split intracellularly by a novel phospho-glycosidase (MupG), constituting the first characterized representative of a novel class of phospho-muramidase enzymes distributed mainly within the *Firmicutes* bacteria.

Hager et al. report on an intriguing mode of attachment used by some bacteria (e.g., *Bacillus anthracis* and *Paenibacillus alvei*) to bind cell-surface proteins to the cell envelope: pyruvylated secondary cell wall polymers act as high-affinity ligands for binding. In this study, the enzymatic pathway leading to the synthesis of pyruvylated disaccharide repeats, [-4-beta-GlcNAc-1,3-(4,6-Pyr)-beta-ManNAc-1-], of the *P. alvei* cell wall polymer was reconstituted. The reconstitution involved recombinant CsaB enzyme, catalyzing the attachment of a pyruvate to position 4 and 6 of ManNAc in the lipid-linked precursor molecule.

Devine provides a concise mini-review about the phosphate starvation regulation in the Gram-negative *E. coli* and the Gram-positive *B. subtilis*. In both organisms, phosphate limitation is sensed by the two-component system PhoPR. However, the mechanisms controlling the Pho response differ. In *Bacillus subtilis*, phosphate-limitation response is linked with wall teichoic acid metabolism. PhoR activity is controlled by biosynthetic intermediates of WTA metabolism, which either promotes or inhibits autokinase activity. In *E. coli*, phosphate is sensed directly through substrate-responsive conformational changes in a phosphate transporter.

Vermassen et al. give a comprehensive overview of the biochemistry and *in vivo* cleavage activity of PG lytic enzymes. This review highlights the “mix and match” approach that many cell wall lytic enzymes have undergone, combining different PG cleavage catalytic functions (e.g., lytic transglycosylase and peptidase activity) within the same enzyme.

The unique chemical nature of PG allows it to act as a potent signaling molecule. Irazoki et al. provide an overview of the process of PG release across a broad range of bacteria and PG sensing by a wide range of hosts. The authors highlight the multiplicity of systems to generate and sense bacterial PG and suggest that there is still a great deal to be learned about the sensing of these important molecules. They conclude that this field will be driven by the development and application of new analytical technologies to identify novel PG receptors.

Peptidoglycan recycling among many Gram-negative bacteria is achieved through a core pathway of degradation, recovery and recycling. In some pathogenic *Neisseria*, the recycling system is partially defective, which leads to an increase in the release of immunostimulatory PG fragments. In their review article, Schaub and Dillard discuss some of the differences between Neisserial PG turnover and other, more intensively studied bacteria such as *E. coli*. They conclude by proposing *Neisseria* sp. as an attractive model system for the study of cell wall growth and turnover due to their lower number of cell wall-active enzymes, variation in cell shape, and natural competence.

In addition to variations in glycan composition and stem-peptide composition, PG can also be O-acetylated at the C-6 of MurNAc, or, less frequently, GlcNAc. Sychanta et al. provide an overview of recent advances in understanding the biochemistry of O-acetyltransferase systems in Gram-positive and Gram-negative bacteria. They also discuss current efforts at understanding the impact of inhibiting these systems and address unanswered biological questions such as the source of acetate for wall modification.

Bacterial cell wall biology remains a major frontier, both in our quest to develop a profound understanding of fundamental microbiology and to discover novel compounds that may be used to treat infections caused by antibiotic resistant bacteria. We hope that this special issue further advances this frontier and inspires additional exploration—peptidoglycan is, in many ways, still as mysterious as it was 7,762 publications ago.

## Author Contributions

All authors listed have made a substantial, direct and intellectual contribution to the work, and approved it for publication.

### Conflict of Interest Statement

The authors declare that the research was conducted in the absence of any commercial or financial relationships that could be construed as a potential conflict of interest.
